# Structural and Functional Loss in Restored Wetland Ecosystems

**DOI:** 10.1371/journal.pbio.1001247

**Published:** 2012-01-24

**Authors:** David Moreno-Mateos, Mary E. Power, Francisco A. Comín, Roxana Yockteng

**Affiliations:** 1 Integrative Biology Department, University of California at Berkeley, Berkeley, California, United States of America; 2 Jasper Ridge Biological Preserve, Stanford University, Woodside, California, United States of America; 3 Department of Conservation of Biodiversity and Ecosystem Restoration, Pyrenean Institute of Ecology – CSIC, Zaragoza, Spain; 4 UMR CNRS 7205, Muséum National d'Histoire Naturelle, Paris, France; McGill University, Canada

## Abstract

Wetlands are among the most productive and economically valuable ecosystems in the world. However, because of human activities, over half of the wetland ecosystems existing in North America, Europe, Australia, and China in the early 20th century have been lost. Ecological restoration to recover critical ecosystem services has been widely attempted, but the degree of actual recovery of ecosystem functioning and structure from these efforts remains uncertain. Our results from a meta-analysis of 621 wetland sites from throughout the world show that even a century after restoration efforts, biological structure (driven mostly by plant assemblages), and biogeochemical functioning (driven primarily by the storage of carbon in wetland soils), remained on average 26% and 23% lower, respectively, than in reference sites. Either recovery has been very slow, or postdisturbance systems have moved towards alternative states that differ from reference conditions. We also found significant effects of environmental settings on the rate and degree of recovery. Large wetland areas (>100 ha) and wetlands restored in warm (temperate and tropical) climates recovered more rapidly than smaller wetlands and wetlands restored in cold climates. Also, wetlands experiencing more (riverine and tidal) hydrologic exchange recovered more rapidly than depressional wetlands. Restoration performance is limited: current restoration practice fails to recover original levels of wetland ecosystem functions, even after many decades. If restoration as currently practiced is used to justify further degradation, global loss of wetland ecosystem function and structure will spread.

## Abstract 2

In restored wetland ecosystems with apparently natural hydrology and biological structure, biogeochemical function may remain degraded, even a century after restoration efforts.

## Author Summary

Wetlands, which include tropical mangroves and boreal peatlands, are among the most valuable ecosystems in the world because they provide critical ecosystem goods and services, such as carbon storage, biodiversity conservation, fish production, water purification, and erosion control. As global change accelerates the loss of wetlands, attempts are increasing to restore this fragile habitat and its associated functioning. There has been no global evaluation, however, of how effective such restoration efforts have been. Here, we present a meta-analysis of the biological structure (driven mostly by plant communities) and biogeochemical functioning (driven primarily by the storage of carbon in wetland soils) of 621 wetland sites. Our analysis suggests that even a century after restoration efforts, these parameters remained on average 26% and 23% (respectively) lower in restored or created wetlands than in reference wetlands. Our results also indicate that ecosystem size and the environmental setting significantly affect the rate of recovery. Recovery may be more likely and more rapid if more than 100 contiguous hectares of habitat are restored. In warm climates, and in settings linked to riverine or tidal flows, recovery can also proceed more rapidly. In general, however, once disturbed, wetlands either recover very slowly or move towards alternative states that differ from reference conditions. Thus, current restoration practice and wetland mitigation policies will maintain and likely accelerate the global loss of wetland ecosystem functions.

## Introduction

From tropical mangroves to boreal peatlands, wetlands are amongst the most productive and economically valuable ecosystems in the world [1]. They provide critical ecosystem goods and services, including carbon storage, biodiversity conservation, fish production, fuel production, water purification, flood and shoreline surge protection and erosion control, and recreation [1]–[3]. However, owing to human activities, over half of the wetland ecosystems existing in the early 20th century have been lost in North America, Europe, Australia, and China [2]. Over the last century, restoration of degraded wetlands and creation of new ones have been attempted, in efforts to recover physical, chemical, and biological processes and entities lost because of wetland destruction or degradation [4]. Frequently, however, this approach does not restore ecosystem structure and functions to preimpact levels [5]–[8]. In North America (including Canada, United States, and Mexico) alone, over US$70 billion have been spent attempting to restore more than 3,000,000 ha of wetlands in the last 20 y (see Text S1) [9], but the recovery trajectories of structure and functions in restored wetlands have not yet been globally assessed [10],[11].

After degradation or natural perturbation, ecosystem structure and functions recover towards reference levels [7],[12], but recovery rates might be affected by the physical characteristics of the ecosystem, the degrading activity, or the environmental setting [7],[12]. Abiotic factors, such as size of restored ecosystems and climate, might affect recovery rates. It could be expected that intensely engineered small (few hectares) wetlands might recover faster than less manipulated, large wetlands (hundreds of hectares) to their original characteristics, but this prediction remains unconfirmed. Higher recovery rates could also be expected in warmer climates than in cold ones, because of accelerated ecosystem processes [7],[13]. Restoration efforts during the recovery process may lead ecosystems to reference states or redirect them towards alternative states [14]–[16] that could also be initiated by prerestoration disturbance itself. If recovery is slow, it could be difficult to distinguish between these alternatives. We surveyed long-term (up to 100 y, available for some but not all of the studied variables) chronosequences of restored wetland ecosystems from 621 restored and created wetlands relative to 556 reference wetlands (Figure S1). Following Article 1.1 of the Ramsar Convention of Wetlands [17], we considered wetlands to be marshes, peatlands, floodplains, mangroves, depressional wetlands, and lacustrine wetlands—submerged permanently or periodically under flowing or still fresh, salty, or brackish water. We compared structure and function of 401 wetlands restored on sites where they had been previously degraded and 220 newly created wetlands (wetland creation de novo is currently accepted for environmental mitigation [4]). We also examined how size of ecosystem and its environmental setting (climate regime and hydrologic connectivity) affected recovery. Using a standardized method (see Materials and Methods), we selected 124 studies (see Text S2) in which ecological responses were measured at known time intervals since restoration. From the selected studies, we extracted 1,501 data points (Tables 1, S1, and S1S2) comparing hydrologic, biological, and biogeochemical variables in restored or created and reference wetlands. Response ratios (see Materials and Methods) were calculated for each data point. Variables selected from the same studies were not necessarily independent (see Materials and Methods), so statistical inferences must be interpreted cautiously.

**Table 1:** Variables measured simultaneously in restored or created and reference wetlands to estimate wetland restoration performance over time.

Wetland Structure and Functions	*n* a	Variables Measured
Hydrology	32	Water level, flooding regime, water storage
Biological components	809	
Vertebrates	166	Abundance, density, species richness, occupancy
Macroinvertebrates	161	Density, abundance, species richness
Plants	439	Plant cover, species richness, biomass, abundance
Biogeochemistry	692	
Carbon storage and cycling	103	Soil total and organic carbon, respiration rate, mineralization rate
Nitrogen storage and cycling	102	Soil total and organic nitrogen, denitrification, and nitrification
Phosphorus storage	103	Soil total and organic phosphorus, Ca-Fe-Al bounded phosphorus
Other elements storage	106	Salinity, soil Fe, Al, Ca, K, Mn, Mg, water dissolved oxygen
Organic matter accumulation	177	Soil organic matter, bulk density, soil texture, soil moisture

Only the most frequently measured variables were included (see Tables S1 and S2, for full description of the variables measuring restoration performance).

a *n* = number of variables used to plot each chronosequence.

We compared recovery trajectories of hydrologic, biological, and biogeochemical variables of restored and created wetlands to address three questions: (a) How fast are biological and biogeochemical components of restored ecosystems changing relative to less perturbed reference ecosystems?; (b) Do these changes trend towards or away from the predisturbed ecosystem or parallel control ecosystems?; and (c) Does wetland size or environmental setting (regional climate, hydrologic connectivity) affect recovery?

## Results/Discussion

### Hydrologic and Biological Recovery

**Figure 1:**
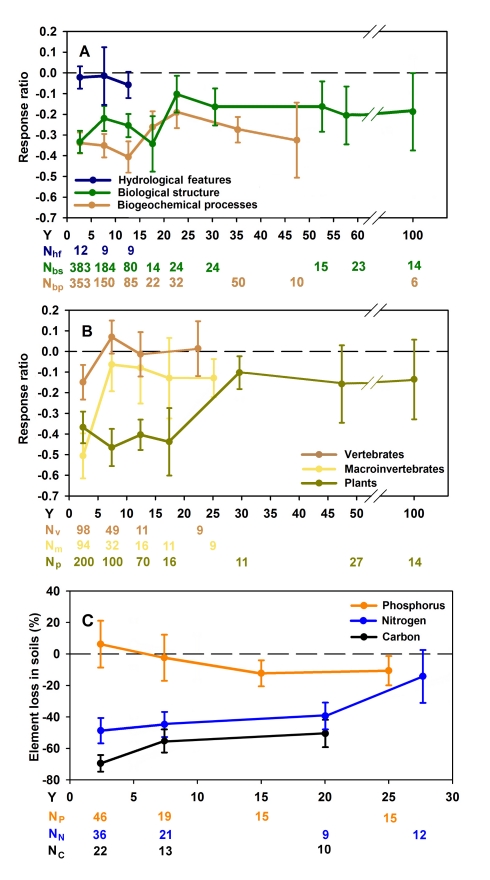
Recovery trajectories of created and restored wetlands. Chronosequences of the means (±standard error [SE]) of the response ratios (see Materials and Methods) of restored and created wetlands at successive age classes of 5 y or 10 consecutive y for hydrology, biological structure, and biogeochemical functions (A) and for the main biological structural components (B). Chronosequences of the means (±SE) of the element loss in soils of restored or created wetlands at successive age classes of 5 y or 10 consecutive y (C). The zero value dashed line represents reference wetlands. Only trend lines for those variables for which we had enough data points (see Materials and Methods) were plotted (N, number of data points used to calculate the mean [±SE] per age class; Y, years after restoration. Subscripts are as follows: bp, biogeochemical processes; bs, biological structure; C, carbon; hf, hydrological features; m, macroinvertebrates; N, nitrogen; p, plants; P, phosphorus; v, vertebrates).

Some hydrologic features can often be restored by manipulating local topography, soil permeability, surface and ground water flows—physical features that are usually engineered in wetland restoration projects. Hydrological features defined for these analyses (Table 1) appeared to be recovered immediately after restoration (Figure 1A), but see Cole [18], Hunt et al. [19], Ahn and Dee [20], and Kumar and Zhao [21] for deeper considerations of challenges to hydrologic restoration in wetlands (from factors like climate variation [20] or complex flow paths of water through heterogeneous vegetation and soils [21]). In addition, all hydrologic variables reported in studies we reviewed were followed only for 10 y to 15 y, so longer-term changes remain unknown.

**Figure 2:**
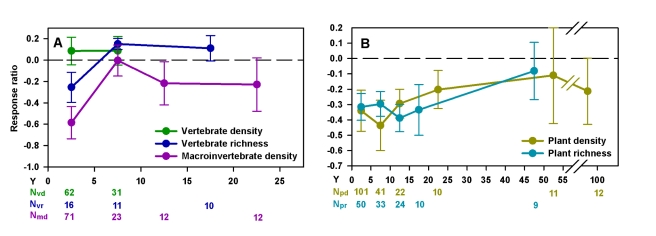
Recovery trajectories of animal and plant richness and density. Chronosequences of the means (±standard error [SE]) of the response ratios (see Materials and Methods) of restored or created wetlands at successive age classes of 5 y or 10 consecutive y for vertebrates and macroinvertebrates density and richness (A) and for plant density and richness (B). Insufficient data points meeting our plotting criteria (see Materials and Methods) were available to plot for macroinvertebrate richness. The zero value dashed line represents reference wetlands (N, number of data points used to calculate the mean [±SE] per age class; Y, years after restoration. Subscripts are as follows: md, macroinvertebrates density; pd, plant density; pr, plant richness; vd, vertebrate density; vr, vertebrates richness).

In contrast to reported hydrologic performance, biological structure (as defined in Table 1) in restored or created wetlands, recovered to only 77% (on average) of reference values (Figure 1A and 1B; Table S3), even 100 y after restoration, when data on 14 taxa from two studies of three wetland sites are available [22],[23]. Abundance, species richness, and diversity of native animals and plants in wetlands were severely reduced following degradation. After restoration, recovery proceeded at different rates, and trajectories plateaued at different levels. Vertebrate assemblages reached similar structural values to those in reference wetlands within 5 y (Figure 1B). Vertebrate richness recovered more slowly than abundance (*p* = 0.021; Figure 2A), possibly reflecting responses by a few highly mobile vertebrate species [24],[25] once hydrological connectivity was restored. Macroinvertebrates (64% noninsects) took 5 y to 10 y to statistically converge with reference assemblages in restored and created wetlands (Figure 1B), and average values never reached absolute reference levels. Many macroinvertebrates cannot recolonize new or restored wetlands by themselves, but are carried in by flowing water or other organisms [26],[27]; however, their short life cycles (often annual or semi-annual) could accelerate population recovery after they arrive [28],[29].

Plant assemblages in restored and created wetlands were slowest to recover. Plants took on average 30 y to converge statistically with reference states; although again, absolute average values of structural features of plant assemblages remained lower than reference levels even after 100 y following restoration (Figures 1B and 2B). The slow and incomplete recovery of plant assemblage might be due to dispersal limitation, vulnerable early life history stages, or sensitivity of any life stage to altered conditions (e.g., reduced organic content of soils, discussed below) during early succession following disturbance [30],[31]. Other factors, such as exotic colonists, subsequent disturbance or altered disturbance regimes, priority effects (historical legacies), and nonlinear interactions may also lead to delayed recovery or persistent differences between restored biota and those in reference wetlands [6],[31],[32].

### Biogeochemical Recovery

Four biogeochemical responses were sufficiently well documented in some studies we reviewed to examine trends over time: these were the storage of carbon, nitrogen, and phosphorus (Figure 1C) (see also storage and cycling combined for carbon and nitrogen in Figure S2A), and the accumulation of organic matter in soil (Figure S2B). The storage and cycling of carbon and nitrogen were drastically reduced from preimpact levels after degradation. In contrast, phosphorus storage seemed unaffected. After restoration, responses were variable. Initially, carbon storage increased slightly but then plateaued below reference levels; nitrogen storage and cycling increased slowly but continuously; and phosphorus storage remained unaffected. Wetland degradation notoriously oxidizes stores of accumulated organic carbon and releases CO_2_ to the atmosphere, as aerobic conditions accelerate microbial respiration [2],. After wetland hydrologic regimes are recovered, more anaerobic conditions allow stores of organic carbon to slowly reaccumulate in the soil. After 20 y, however, carbon storage in restored and created wetland soils was still significantly lower (by 50%; *p* = 0.008) than in reference wetlands (Figure 1C; Table S3; Text S1; data from six studies of 21 wetlands) Organic matter accumulated slowly [34],[35], so that average values remained only 62% of the value at the reference wetlands 20–30 y following restoration (Figure S2B; data from seven studies of 21 wetlands).

Aerobic conditions in degraded wetlands also perturb nitrogen storage and cycling, allowing mineralization of organic N and transformation of ammonium to nitrate [2]. Nitrate is quickly processed by microorganisms and plants, leaving the original pool of nitrogen in the soil depleted or unavailable. Nitrogen storage remained significantly lower in restored wetlands for 30 y after the wetlands were restored or created (Figure 1C; Table S3). Depleted or unavailable soil nitrogen can limit wetland productivity, retarding carbon storage [33],[36]. In contrast, total phosphorus decreased only slightly in restored or created wetlands and did not show significant differences with reference wetlands (Figure 1C). Although, phosphorus chemical fractions could change in representation, the amount of total phosphorus did not change significantly [37]. This lack of variation in phosphorus might be explained because of the more conservative cycling by phosphorus (lack of exchange with the atmosphere) [38]. In addition, without extrinsic inputs, phosphorus levels would be geologically determined.

After 50 y to 100 y, restored wetlands recovered only to an average of 74% of their biogeochemical functioning relative to reference wetlands (Figure 1A; data from two studies of seven wetlands; data of wetlands recovering for more than 50 y after restoration were not plotted in Figure 1A because the sample size did not meet our criteria for average points, see Materials and Methods section, on this graph). Since phosphorus storage appeared only slightly changed, the overall lack of recovery of biogeochemical functioning may have been driven largely by the low recovery of the carbon storage and the low accumulation of soil organic matter (see Text S1).

### Effects of Size and Environmental Setting

Comparing wetland recovery trajectories under different conditions may shed light on factors that impede or facilitate recovery. Although biogeochemical responses in both restored and created wetlands were similar, biological structure in created wetlands approached reference conditions more quickly (Figure S3A and S3B; Table S5). Created wetlands may have been engineered to force the initial system towards defined reference conditions [39].

**Figure 3:**
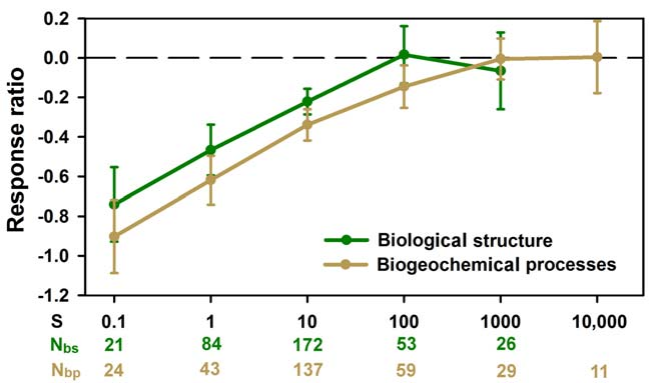
Effect of size on wetland recovery. Evolution of the mean (±standard error [SE]) of the response ratios (see Materials and Methods) of restored or created wetlands at successive size categories for wetlands between 0 y to 5 y after restoration or creation. The zero value dashed line represents reference wetlands. Mean (±SE) at 0.1 ha was estimated for wetlands with sizes ≤0.1 ha. Means (±SE) at 1 ha were estimated for wetlands in which sizes ranged between 0.1 ha and 1 ha. The same approach was used to estimate the means (±SE) at 10, 100, 1,000, and 10,000 ha (N, number of data points used to calculate the mean [±SE] per age class; size, size in hectares of the restored wetlands. Subscripts are as follows: bp, biogeochemical processes; bs, biological structure).

Ecosystem size and local and regional context affect wetland recovery. Large wetlands (>100 ha) appeared to recover their biological structure and biogeochemical functions sooner after restoration or creation than smaller wetlands (Figures 3 and S4; Table S4; data from 13 studies of 25 wetlands). This differential recovery suggests that small wetlands may not provide adequate local resources or connectivity for local biota to restore preimpact functioning. Restored and created wetlands, particularly if small, may have become more isolated and surrounded by more fragmented landscapes than they had been before impact [40]. Also, small wetlands would only be able to support a limited number of individuals, and thus, will not be able to support all the species, particularly taxa with large body sizes, formerly capable of occupying the area [41].

**Figure 4:**
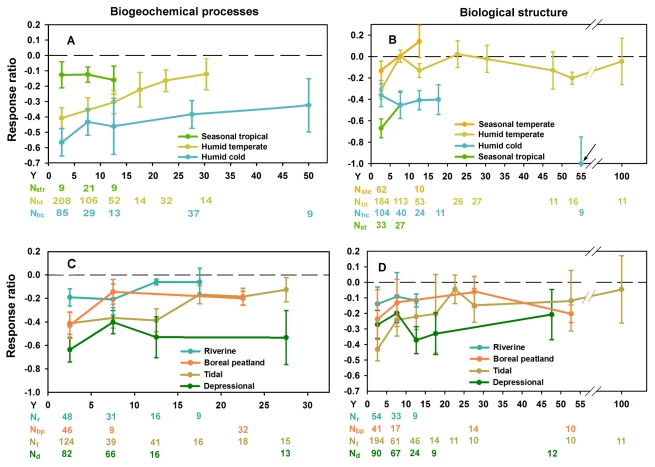
Effects of climate and hydrology on wetland recovery trajectories. Chronosequences of the means (±standard error [SE]) of the response ratios (see Materials and Methods) of restored and created wetlands at successive age classes of 5 y or 10 consecutive y for biogeochemical functions and for biological structures under contrasting climates (A and B), and under different hydrologic connectivity (C and D) [31]. The zero value dashed line represents reference wetlands. The arrow (B) indicates the outlier mean value of two restoration studies with extremely low recovery rates (N, number of data points used to calculate the mean [±SE] per age class; Y, years after restoration. Subscripts are as follows: bp, boreal peatland; d, depressional; hc, humid cold; ht, humid temperate; r, riverine; str, seasonal tropical; ste, seasonal temperate; t, tidal).

Regional climate had a strong effect on the sequence and rate of wetland recovery following restoration. As expected, warm temperatures accelerate ecosystem processes [7],[13],[42], including those mediating biological and biogeochemical recovery after wetland restoration or creation. In tropical and summer-warm temperate climates, wetlands approached reference conditions relatively rapidly, while wetlands restored in cold climates had not recovered to reference conditions after 50 y (Figure 4A and 4B; Tables S3 and S5). In tropical climates only, biogeochemical variables recovered to reference levels before biological structure did (data from eight studies of eight wetlands). Whether this difference in recovery sequence is a real aspect of tropical wetlands, or an artifact of small sample size, remains to be seen. In a much larger sample of studies from temperate climates this sequence was reversed, and biogeochemical recovery was slower. Biological structural variables appeared recovered 5 y after restoration, while even 30 y after restoration, biogeochemical functions had only recovered to 79% of reference levels (data from 83 studies of 302 wetlands). In cold climates, corresponding biogeochemical recovery was only 53% 50 y after restoration; both biogeochemical functions and biological structure variables remained statistically distinct from reference conditions for the entire (50-y) chronosequence (Figure 4A and 4B; Tables S3 and S5; data from 33 studies of 311 wetlands).

Hydrologic setting [43] also affected recovery (Figure 4C and 4D; Tables S3 and S5). Riverine and tidal wetlands, linked to larger hydrologic regimes by natural flow variation, recovered biogeochemical functions and biological structure after 20 y and 30 y, respectively (data from 73 studies of 210 wetlands). These results are similar to those (15 y to 25 y to recover the original biotic composition and diversity) found by Borja et al. [8] in 51 globally distributed estuarine and coastal ecosystems. In contrast, wetlands in inland depressions that were watered by precipitation or groundwater flow had not recovered to reference conditions even after 50 y following restoration (data from 36 studies of 358 wetlands). Peatlands (usually only the upper layer [<1 m] of peat was removed) recovered biological structure immediately, but 30 y after restoration, biogeochemical functioning in peatlands remained statistically lower than in reference wetlands (data from 11 studies of 18 wetlands).

### Slow Recovery or Alternative States?

Two hypotheses could explain the lag in biological and biogeochemical recovery of the biological structure and biogeochemical functioning. First, the chronosequences we examined may be too short (<30 y) for full recovery, especially of carbon and nitrogen storage [44]. Second, restored wetlands may have shifted to alternative states, different from their condition before degradation [14],[15]. The subreference plateaus of soil organic accumulation, carbon storage, and general biogeochemical functioning could support the second hypothesis of alternative states in restored systems. Slow recovery of plant density and richness might be linked to lags in carbon storage. Mutualist symbionts critical for plant productivity (e.g., N-fixing bacteria [2] or mycorrhizal fungi [45]) may be absent in recently (<50 y) restored wetland soils. Alternatively, fast-growing, early successional terrestrial plants, and potentially also wetland plants, usually allocate most of their carbon to photosynthetically active structures of low density and high nutrient content, which are easily grazed or rapidly decomposed, retarding local storage of carbon [46],[47].

### Comparison with Other Findings

Two other studies have assessed recovery rates of large scale natural ecosystems following disturbance or perturbations [7],[12]. Both of these studies examined a broad range of ecosystem types (terrestrial, freshwater, and marine), including wetlands. Jones and Schmitz [12] found that across ecosystems and perturbation types (natural and human-caused), about half of the tracked response variables were considered by original authors to have recovered to preimpact states. Jones and Schmitz computed averaged recovery times for the subsamples of variables and cases that primary authors considered to have recovered over the course of their studies. These recovery times ranged from about 10 y to 40 y, and were longer for forests, and following human-caused, rather than natural perturbations. To assess whether systems had recovered or not, Jones and Schmitz used authors' expert opinion, return to historic initial conditions, or approach to parallel reference states (our study evaluated recovery only for studies using the last of these criteria). Given the narrower scope of our study (assessing wetlands only), and our different analysis approach, estimated recovery times from these two reviews are surprisingly similar. Rey Benayas et al. [7] studied recovery across a wide range of human-perturbed ecosystems, including wetlands. Using (as we did) the response ratio of restored to reference ecosystems, Benayas et al. found biodiversity and selected ecosystems services to be 86% and 80% recovered in a sample of 89 cases pooled over all age categories since perturbation. Interestingly, they reported slightly (6%) higher recovery in biological variables compared to ecosystems services (nutrient cycling; primary production; provisioning of timber, fish, and food crops; and regulation of climate, water supply, and soil). These ecosystem services overlap in part with categories of biogeochemical variables in our study (e.g., carbon and nutrient storage and cycling). The similarity between their results and our finding (that biological variables were 9% more recovered than these biogeochemical responses) suggests that structural recovery might often be necessary to achieve functional recovery.

### Conclusions

Our meta-analysis suggests that recovery of wetlands following restoration as currently practiced is often slow and incomplete. In warm climates, and in settings linked to riverine or tidal flows, recovery may proceed more rapidly. Recovery may also be more likely and more rapid if >100 contiguous ha are restored. In many wetlands, however, ecosystem services may not be fully recovered even when wetlands appear to be biologically restored. If markets for ecosystem services and mitigation offsets from restored or created wetlands are used to justify further wetland degradation, net loss of global wetland services will continue and likely accelerate (see also Race and Fonseca [48]). We join other wetland ecologists and restoration scientists in calling for better scientific understanding of biotic and abiotic factors that constrain ecosystem restoration. For our common future, we need more realistic, long-term evaluations to find better ways to alleviate constraints limiting the recovery of wetland ecosystems.

## Materials and Methods

### Literature Search

On the 22nd of December 2010 a reference search was done in the scientific database ISI Web of Science – SCI-Expanded. The terms used were “(wetland* or floodplain*or peatland* or marsh* or mangrove*) same (restor* or creat* or re-creat* or rehabilit*).” We used these terms to cover a wide variety of wetlands as defined in the Article 1.1 of the Ramsar Convention text [7]. For this analysis, we considered restored wetlands to be wetlands recreated on sites where wetlands had formerly existed but been drained or otherwise severely degraded. Created wetlands were described by authors as wetlands built on sites that lacked previous wetland history. We selected studies of wetlands under natural hydrological regimes, planted with native species, and in which no allochthonous substrates were imported during the restoration or creation activities. For this reason, the term “construct*” was not included in the search terms, because we found in an independent search that >99% of the studies of constructed wetlands were of highly artificial systems not maintained under natural conditions. The search produced 2,959 selected articles. We applied the general selection criterion: “Articles must compare measurements of structural components and biogeochemical processes in restored or created and reference wetlands at a known age.” Under this criterion we selected 172 articles. These articles were read, and those in which data were averaged over time intervals larger than 5 y, those in which sizes differing by more than one order of magnitude were averaged, and those lacking reliable measurements or comparable restored and reference conditions were discarded, leaving 124 articles (see Text S2). Reference wetlands were usually adjacent to restored or created wetlands, although in some cases they were separated by several kilometers (maximum distance found was ∼100 km). In all cases, restored or created wetlands were of the same wetland hydrogeomorphic type [17] as reference wetlands with which they were compared. From the selected articles, six were carried out on experimental wetlands, the rest were carried out on wetland restoration or creation projects. Studies either described measurements at a known age after wetlands were restored or created, or a chronosequence of the progression during the wetland restoration process. Restored and created wetlands were located in 12 countries and totaled >21,294 ha in area and reference wetlands >19,694 ha. The exact total area is not known because it was not reported in 23 out of the 124 selected studies.

### Data Extraction

Measurements of structural components and biogeochemical processes were extracted from the main text, tables, and figures of the articles. When abundance of one species was measured at different life stages, only the adult abundance of each species was selected. Variables describing hydrological structure, biological structure, element storage and cycling, and organic matter accumulation were classified as structural components or biogeochemical processes according to wetland functions described by Smith et al. [42], and as ecosystem services described in the Millennium Ecosystem Assessment (MEA) (organic matter accumulation was sometimes designated as “soil formation” in the MEA but not in other soil science references) [49].

Element storage and cycling variables measured processes (mineralization or denitrification) and concentration of elements in different pools (total content in soil, organic content in soil, or content in roots), which suggest how nutrients are moving between pools through biotic and abiotic processes (Tables S1 and S2). The studies presented enough data points to plot recovery of storage of carbon, nitrogen, and phosphorus.

### Response Ratio Calculation

To standardize and compare data, we used standard response ratios used in meta-analysis, *ln*(*Xrest*+1/*Xref*+1) [3], where *Xrest* is the value of the measured variable in the restored or created wetland and *Xref* is the value of the measured variable in the reference wetland. To avoid the value “0” in the natural logarithm of the equation, “1” was added to both values in restored or created and reference wetlands. The effect of adding “1” to the values in the response ratio equation has been demonstrated to have little effect on conclusions [50]. The effect size was not weighted because variance was reported for only 64% of the variables. Differences between weighted and unweighted meta-analysis statistics are generally small [7].

As variables depicting structural components and biogeochemical processes in restored or created wetlands converged to values in the reference wetlands, recovery of function was generally enhanced. But for some variables, such as soil bulk density [51],[52], or proportions of exotic species [53],[54], higher values are associated with lower levels of wetland recovery. In some cases, the specific context of a study made variables negative for recovery of a particular restored wetland, e.g., the presence of woody species where none had occurred in the reference wetlands [55],[56]. In these cases (11% of the collected variables), we changed the sign to reverse the value of the response ratio.

### Data Classification

For each variable we recorded the age of the restored or created wetland, the wetland hydrogeomorphic type, the number of restored or created and reference wetlands considered in a given study, the size (ha) of the restored or created and reference wetlands, the initial condition (restored or created), the geographic location, and the climate. Most data (49%) were from wetlands that had been restored or created for less than 5 y (Figure S1). If data from several wetlands of different sizes were averaged in the study, then we also averaged the sizes for our analysis. The geographic location was registered as the latitude and longitude in degrees of the center of the wetland or group of wetlands. The climate was classified according to the last revision of the Köppen-Geiger climate classification [57]. We used the name humid temperate climate for Cf climate, humid cold climate for Df climate, seasonal temperate climate for Cs climate (with dry summer), and seasonal tropical for A climates. Two of our sampled studies were done in seasonal temperate climate with dry winter (Köppen-Geiger climate classification Cw), and were not considered in our climate study. Wetland hydrogeomorphic type was classified according to Brinson [58] and Smith et al. [42] as depressional, riverine, tidal, peatland, lacustrine, and seeping slope. Only three studies were on lacustrine wetlands and one on seeping slope wetlands, so these types were not considered in our study of differences among wetland types.

In studies where more than one wetland was studied and data were available for each individual wetland, data were collected for each wetland. In 27 studies, more than one wetland was compared with the same reference wetland, and in 11 studies, restored or created wetlands were compared with more than one reference wetland. All studies where more reference rather than restored or created wetlands were studied provided only averaged data for both groups of wetlands. We calculated contingency tables between the wetland size, the initial conditions (created versus restored), and the covariates included in the environmental setting section (climate and wetland hydrogeomorphic type), using contingency coefficients (*C*), to test for independence between them. Wetland type showed relevant degrees of association with the climate (*C* = 0.63) and wetland size (*C* = 0.58), the rest of variables had coefficients below 0.5, indicating low degree of association. These associations may be explained by the influence of the climate on wetland types, e.g., peatlands are usually associated to cold climates, and mangroves to tropical climates. Also, peatlands usually extend over vast surfaces (hundreds or thousands of hectares) and depressional wetlands are usually small basins (less than 10 ha or few tens of hectares).

### Statistical Analysis

Because data were non-normally distributed (according to the Kolmogorov-Smirnoff test for normality), we used Wilcoxon signed rank tests to test for significant deviations from zero (no difference from reference conditions) for each estimated mean of the response ratios for variables at each age interval of a restored or created wetland. To test for differences between the same variable measured under two different environmental settings at a given recovery time, we used Kruskal-Wallis tests.

### Chronosequences Plotting

To plot the temporal trends, the mean values and the standard error of each variable with every age class of 5 y (0–4.9, 5–9.9, etc) were used. The criterion for a mean for a certain age class to be used in the plot was that it must have been derived from at least nine different data points obtained from at least two different studies. When this criterion was not fulfilled, the mean values and standard error of age classes of 10 y (e.g., 10–19.9, or 20–30) were used. Temporal trend lines were fitted when enough data to calculate means for two or more age classes were available.

## Supporting Information

Figure S1 **Distribution of wetland sizes across wetland ages for the 654 restored and created wetlands considered in the study.**
(TIF)

Figure S2 **Chronosequences for the storage and cycling of carbon and nitrogen (A), and for the accumulation of organic matter in soils (B).**
(TIF)

Figure S3 **Chronosequences for biogeochemical processes (A) and for biological structures (B) under contrasting initial conditions (restored wetlands versus wetlands created de novo in dry lands).**
(TIF)

Figure S4 **Evolution of the response ratios of restored or created wetlands at successive size categories for wetlands between 5 y to 15 y after restoration or creation.**
(TIF)

Table S1 **Variables measuring structural components.**
(DOC)

Table S2 **Variables measuring biogeochemical processes.**
(DOC)

Table S3 **Statistical significance of differences between the means of the response ratios in restored or created versus reference wetlands.**
(DOC)

Table S4 **Statistical significance of differences between the means of the response ratios in restored or created versus reference wetlands at each size interval.**
(DOC)

Table S5 **Statistical significance of differences between the response ratios in restored or created wetlands under different environmental settings.**
(DOC)

Text S1 **Wetland restoration investment and carbon storage calculation.**
(DOC)

Text S2 **References used in the meta-analysis.**
(DOC)
