# Supplementary figures and images for: Structural and Functional Loss in Restored Wetland Ecosystems
Source: PLoS Biol. 2012 Jan 24;10(1):e1001247. doi: 10.1371/journal.pbio.1001247 (PMC3265451; doi:10.1371/journal.pbio.1001247)

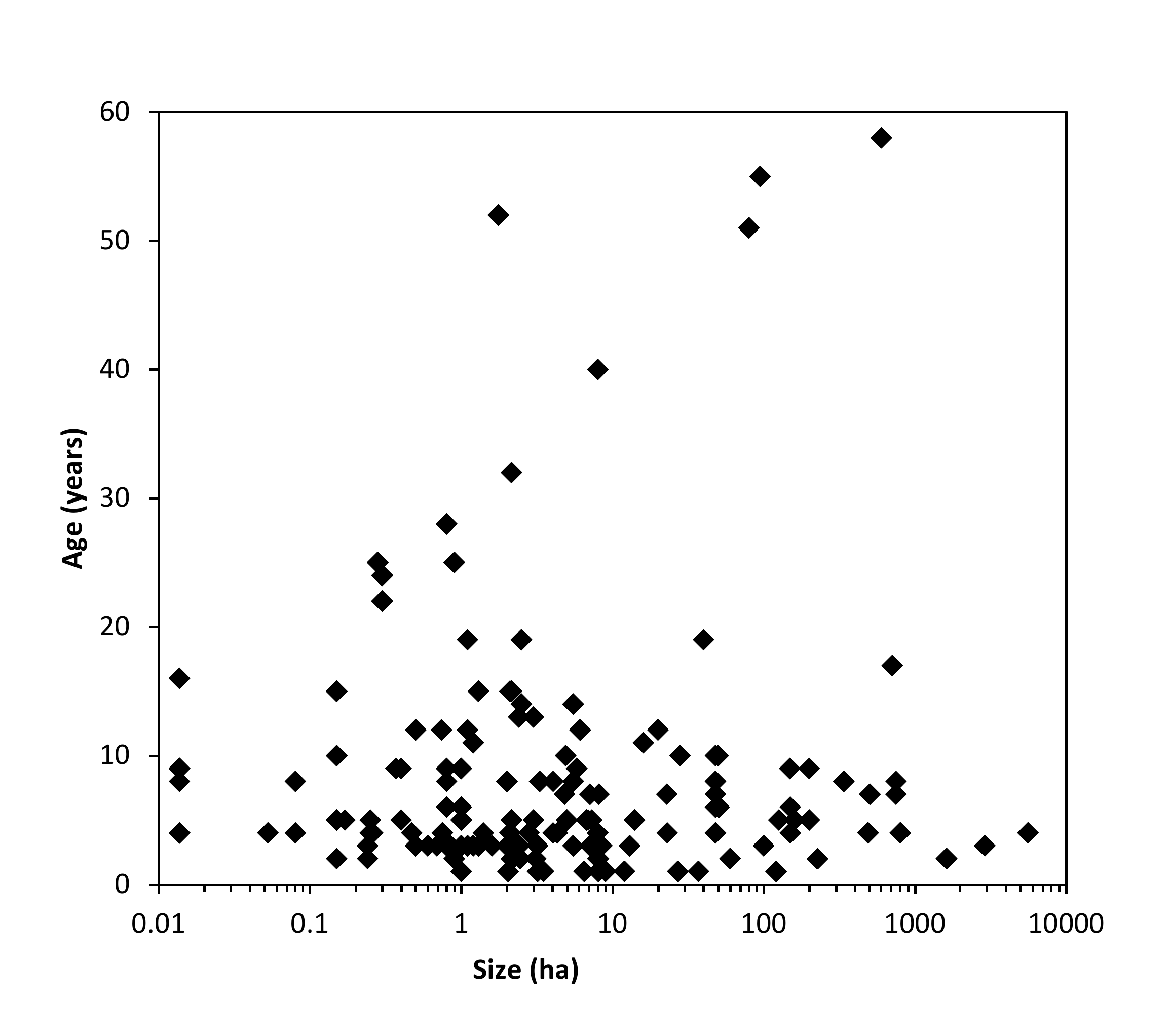

Supplement: Figure S1 — Distribution of wetland sizes across wetland ages for the 654 restored and created wetlands considered in the study. (TIF) [file pbio.1001247.s001.tif]

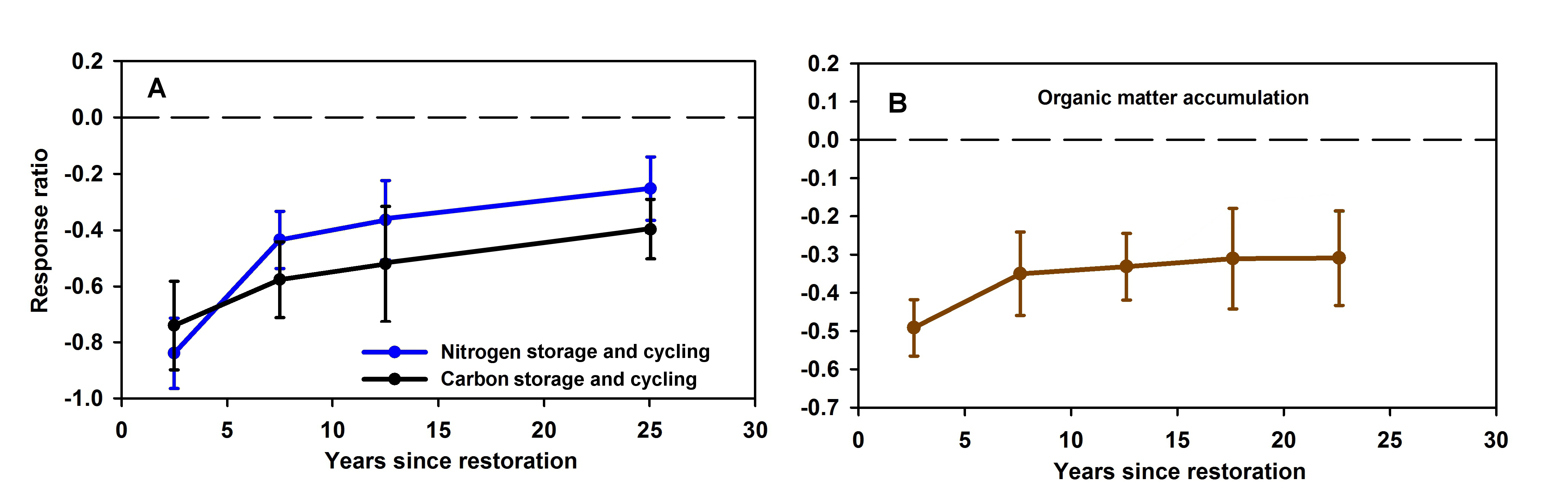

Supplement: Figure S2 — Chronosequences for the storage and cycling of carbon and nitrogen (A), and for the accumulation of organic matter in soils (B). (TIF) [file pbio.1001247.s002.tif]

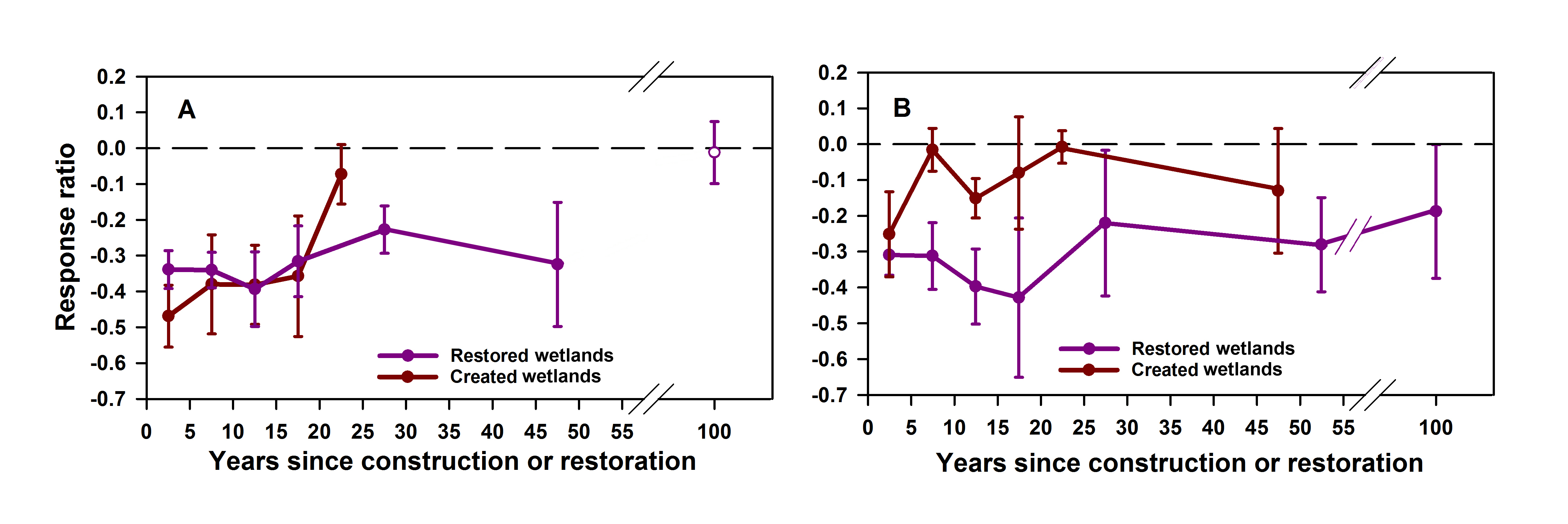

Supplement: Figure S3 — Chronosequences for biogeochemical processes (A) and for biological structures (B) under contrasting initial conditions (restored wetlands versus wetlands created de novo in dry lands). (TIF) [file pbio.1001247.s003.tif]

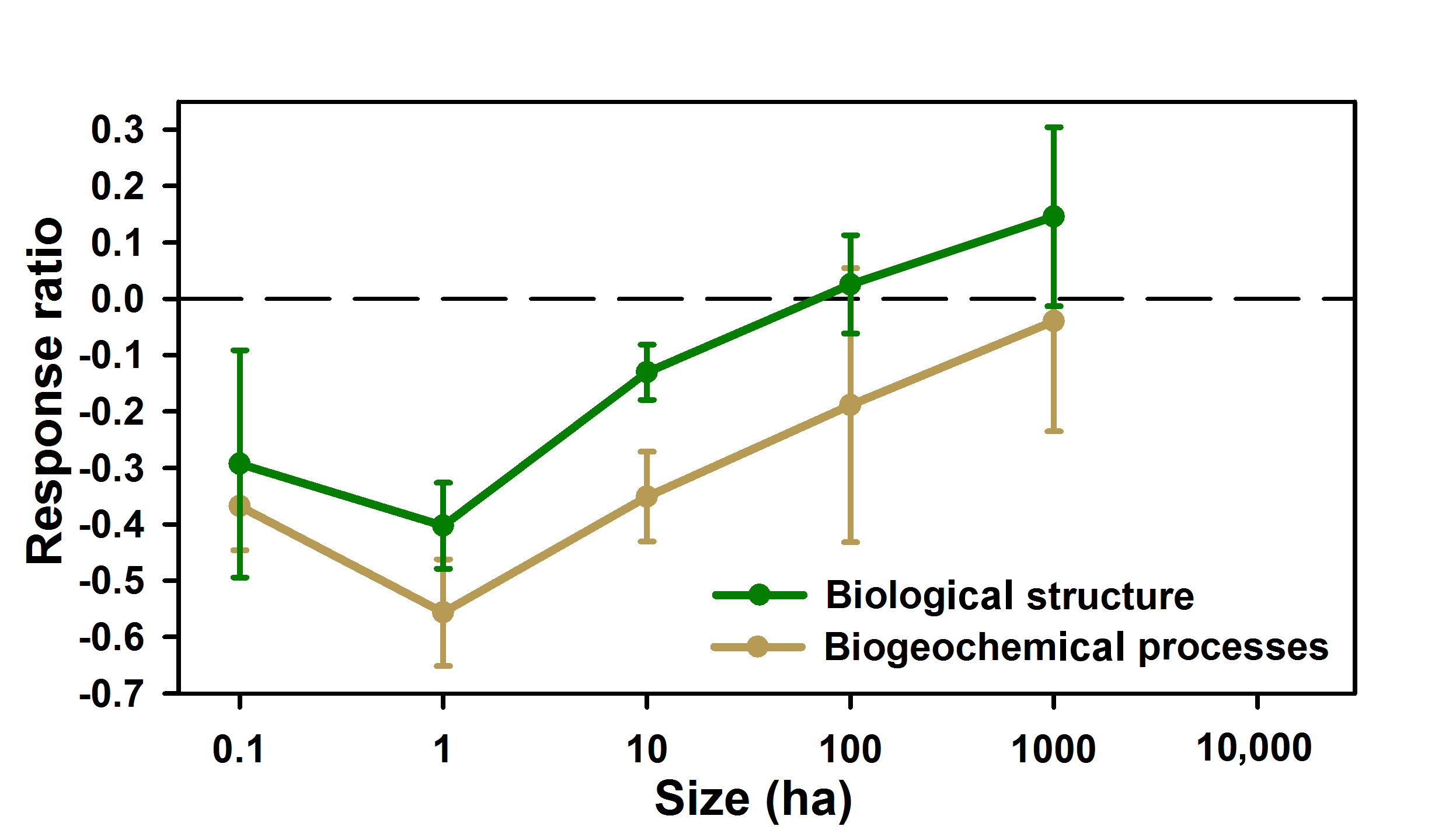

Supplement: Figure S4 — Evolution of the response ratios of restored or created wetlands at successive size categories for wetlands between 5 y to 15 y after restoration or creation. (TIF) [file pbio.1001247.s004.tif]
