# Supplementary material for: Structural and Functional Loss in Restored Wetland Ecosystems
Source: PLoS Biol. 2012 Jan 24;10(1):e1001247. doi: 10.1371/journal.pbio.1001247 (PMC3265451; doi:10.1371/journal.pbio.1001247)
Supplement: Table S1 — Variables measuring structural components. (DOC) [file pbio.1001247.s005.doc]

**Table S1**. Variables measuring structural components (n=809) simultaneously in restored or created and reference wetlands to estimate wetland restoration performance along a 100-years chronosequence. The last column (n) indicates the number of variables used in the analysis representing more than 5% of the total number of variables (N=number of variables used to plot the chronosequence).

| **Hydrological structure (N=34)** | **Units** | **n** |
| --- | --- | --- |
| Water level | m | 12 |
| Flooding regime | unitless index | 10 |
| Water storage | unitless index | 6 |
| **Biological structure (N=775)** |  |  |
| **Vertebrates (N=166)** |  |  |
| Density | individuals/area or sample unit | 42 |
| Abundance | individuals/wetland | 41 |
| Absolute species richness | no. spp/wetland | 29 |
| Relative abundance | % spp of a functional or taxonomical group | 18 |
| Occupancy | % of ecosystem units occupied by one species | 11 |
| Diversity | Shannon-Wiener index | 10 |
| Species richness per area unit | no. spp/area | 6 |
| **Macroinvertebrates (N=161)** |  |  |
| Density | Individuals/area or sample unit | 102 |
| Relative abundance | % spp of a functional or taxonomical group | 24 |
| Absolute species richness | no. spp/wetland | 12 |
| Abundance | individuals/wetland | 9 |
| Species richness per sample or area unit | no. spp/sample or area | 8 |
| Diversity | Shannon-Wiener index | 5 |
| **Plants (N=439)** |  |  |
| Plant cover | % | 141 |
| Plant species richness | no. spp/wetland | 87 |
| Plant biomass | g/m^2^ | 75 |
| Abundance | stems or individuals/area | 19 |
| Diversity | Shannon-Wiener index | 15 |
| Stem height | m | 15 |
| Basal area | m^2^/ha | 11 |
| Seed density | seeds/m^2^ of soil | 7 |
| Species richness per area or sample unit | no. spp/sample or area | 6 |
