# Supplementary material for: Structural and Functional Loss in Restored Wetland Ecosystems
Source: PLoS Biol. 2012 Jan 24;10(1):e1001247. doi: 10.1371/journal.pbio.1001247 (PMC3265451; doi:10.1371/journal.pbio.1001247)
Supplement: Table S2 — Variables measuring biogeochemical processes. (DOC) [file pbio.1001247.s006.doc]

**Table S2**. Variables measuring biogeochemical functions (n=692) simultaneously in restored or created and reference wetlands to estimate wetland restoration performance along a 100-year chronosequence. The last column (n) indicates the number of variables used in the analysis representing more than 5% of the total number of variables. (N=number of variables used to plot the chronosequence).

| **Elements storage and cycling (N=471)** | **Units** | **n** |
| --- | --- | --- |
| **Carbon storage and cycling (N=103)** |  |  |
| Soil organic C | mg C/g soil or g C/m^2^ soil or % | 23 |
| Soil total C | mg C/g soil or g C/m^2^ soil or % | 23 |
| Soil C:N | wt (g):wt (g) | 16 |
| Respiration | gC/g soil or m^2^/time | 15 |
| C mineralization rate | µmol CO_2_/g soil/s or g/m^2^·day | 10 |
| Root C content | g C/m^2^ soil | 5 |
| **Nitrogen storage and cycling (N=102)** |  |  |
| Soil total N | mg N/g soil or g N/m^2^ soil or % | 47 |
| Soil organic NO3 and NH4 | μg/cm^3^ or μg/g | 29 |
| Denitrification and nitrification | ng CO2/cm^3^·hr | 11 |
| **Phosphorus storage (N=103)** |  |  |
| Soil total P | mg P/g soil or μg P/cm3 or g P/m^2^ soil or % | 53 |
| Soil PO4 | mg P/g soil or μg P/cm3 | 16 |
| Soil organic P | μg P/cm3 or mg P/g soil | 16 |
| Soil Ca, Fe, Al bounded P | μg P/cm3 or mg P/g soil | 9 |
| **Other elements storage (N=106)** |  |  |
| Salinity and conductivity | ‰ or % or μg | 19 |
| Several forms of soil Fe | kg/ha or kmol/ha | 14 |
| Several forms of soil Ca | mg/kg | 13 |
| Dissolved oxygen | mg/L | 12 |
| Several forms of soil K | mg/kg | 12 |
| Several forms of soil Al | kg/ha or kmol/ha | 11 |
| Several forms of soil Mg | mg/kg | 9 |
| Several forms of soil Mn | kmol/ha or mg/kg | 7 |
| **Organic matter accumulation (N=177)** |  |  |
| Soil organic matter | g C/m^2^ soil or % | 64 |
| Bulk density | g/cm^3^ | 35 |
| Soil texture | % | 21 |
| Soil moisture | g/cm^3^ | 13 |
| Soil porosity | % | 6 |
