# Supplementary material for: Structural and Functional Loss in Restored Wetland Ecosystems
Source: PLoS Biol. 2012 Jan 24;10(1):e1001247. doi: 10.1371/journal.pbio.1001247 (PMC3265451; doi:10.1371/journal.pbio.1001247)
Supplement: Table S3 — Statistical significance of differences between the means of the response ratios in restored or created versus reference wetlands. (DOC) [file pbio.1001247.s007.doc]

**Table S3.** Statistical significance of differences between the means of the response ratios in restored or created versus reference wetlands at each age class in years (Wilcoxon ranked sign test)(nd = no data available, Proc.= processes, * this age class comprises 10 years starting at the beginning of the interval and ending at the end of the interval of the next age class)

|  |  | **Years since restoration or creation (*p* values)** | | | | | | | | | |
| --- | --- | --- | --- | --- | --- | --- | --- | --- | --- | --- | --- |
| **Figure** | **Described variable** | **0-5** | **5.1-10** | **10.1-15** | **15.1-20** | **20.1-25** | **25.1-30** | **30.1-35** | **50-55** | **55.1-60** | **100** |
| 1A | Biogeochemical proc. | 0.000 | 0.000 | 0.000 | 0.001 | 0.011 | nd | 0.000* | 0.023 | nd | nd |
| 1A | Biological structure | 0.000 | 0.000 | 0.000 | 0.023 | 0.324 | 0.052* | nd | 0.054 | 0.009 | 0.255 |
| 1A | Hydrological structure | 0.395 | 0.500 | 0.155 | nd | nd | nd | nd | nd | nd | nd |
| 1B | Macroinvertebrate assemblage | 0.000 | 0.233 | 0.139 | 0.249 | 0.062* | nd | nd | nd | nd | nd |
| 1B | Plant assemblage | 0.000 | 0.000 | 0.000 | 0.010 | nd | 0.221* | nd | 0.096 | nd | 0.377 |
| 1B | Vertebrate assemblage | 0.186 | 0.268 | 0.288 | 0.444 | nd | nd | nd | nd | nd | nd |
| 1C | Carbon storage | 0.000 | 0.001 | nd | 0.008* | nd | nd | nd | nd | nd | nd |
| 1C | Nitrogen storage | 0.001 | 0.001 | nd | 0.008* | nd | 0.440 | nd | nd | nd | nd |
| 1C | Phosphorus storage | 0.249 | 0.687 | 0.125* | nd | nd | 0.345* | nd | nd | nd | nd |
| 3A | Biogeochemical proc. seasonal tropical | 0.057 | 0.020 | 0.046 | nd | nd | nd | nd | nd | nd | nd |
| 3A | Biogeochemical proc. humid temperate | 0.000 | 0.000 | 0.000 | 0.025 | 0.000 | 0.181* | nd | nd | nd | nd |
| 3A | Biogeochemical proc. humid cold | 0.00 | 0.000 | 0.037 | nd | nd | 0.000 | nd | 0.043 | nd | nd |
| 3B | Biological structure seasonal tropical | 0.000 | 0.001 | nd | nd | nd | nd | nd | nd | nd | nd |
| 3B | Biological structure seasonal temperate | 0.118 | nd | 0.124 | nd | nd | nd | nd | nd | nd | nd |
| 3B | Biological structure humid temperate | 0.000 | 0.340 | 0.004 | nd | 0.424 | 0.281* | nd | 0.297 | 0.002 | 0.464 |
| 3B | Biological structure. humid cold | 0.000 | 0.000 | 0.000 | 0.010 | nd | nd | nd | nd | nd | nd |
| 3C | Biogeochemical depressional | 0.000 | 0.000 | 0.004 | nd | nd | 0.014 | nd | nd | nd | nd |
| 3C | Biogeochemical riverine | 0.002 | 0.058 | 0.007 | 0.249 | nd | nd | nd | nd | nd | nd |
| 3C | Biogeochemical tidal | 0.000 | 0.000 | 0.000 | 0.018 | 0.016 | 0.276 | nd | nd | nd | nd |
| 3C | Biogeochemical peatland | 0.000 | nd | nd | nd | nd | 0.000 | nd | nd | nd | nd |
| 3D | Biological structure depressional | 0.000 | 0.010 | 0.000 | 0.018 | nd | nd | nd | nd | nd | nd |
| 3D | Biological structure riverine | 0.160 | 0.459 | 0.010 | nd | nd | nd | nd | nd | nd | nd |
| 3D | Biological structure tidal | 0.000 | 0.010 | 0.002 | 0.172 | 0.429 | 0.107 | nd | 0.254 | nd | 0.464 |
| 3D | Biological structure peatland | 0.107 | 0.230 | nd | nd | 0.285 | nd | nd | 0.018 | nd | nd |
| S2A | Carbon storage and cycling | 0.000 | 0.000 | 0.009 | nd | 0.002* | nd | nd | nd | nd | nd |
| S2A | Nitrogen storage and cycling | 0.000 | 0.000 | 0.003 | nd | 0.015* | nd | nd | nd | nd | nd |
| S2A | Phosphorus storage | 0.123 | 0.326 | 0.032 | nd | 0.050* | nd | nd | nd | nd | nd |
| S2B | Accumulation organic matter | 0.000 | 0.000 | 0.000 | 0.021 | 0.010 | nd | nd | nd | nd | nd |
| S4A | Biogeochemical proc. restored | 0.000 | 0.000 | 0.000 | 0.000 | nd | 0.000 | nd | 0.014 | nd | nd |
| S4A | Biogeochemical proc. created | 0.000 | 0.001 | 0.000 | 0.014 | 0.036 | nd | nd | nd | nd | nd |
| S4B | Biological structure restored | 0.000 | 0.000 | 0.000 | 0.019 | nd | 0.081 | nd | 0.002 | nd | 0.256 |
| S4B | Biological structure created | 0.004 | 0.449 | 0.003 | 0.297 | 0.500 | nd | nd | 0.254 | nd | nd |
