# Supplementary material for: Structural and Functional Loss in Restored Wetland Ecosystems
Source: PLoS Biol. 2012 Jan 24;10(1):e1001247. doi: 10.1371/journal.pbio.1001247 (PMC3265451; doi:10.1371/journal.pbio.1001247)
Supplement: Table S4 — Statistical significance of differences between the means of the response ratios in restored or created versus reference wetlands at each size interval. (DOC) [file pbio.1001247.s008.doc]

**Table S4. Statistical significance of differences between the means of the response ratios in restored or created versus reference wetlands at each size interval (Wilcoxon ranked sign test)(nd = no data available)**

|  |  | **Area (ha) (*p* values)** | | | | | |
| --- | --- | --- | --- | --- | --- | --- | --- |
| **Figure** | **Described variable** | **0-0.1** | **0.11-1** | **1.1-10** | **10.1-100** | **101-1000** | **1001-10,000** |
| 2 | Biogeochemical processes | 0.000 | 0.000 | 0.000 | 0.034 | 0.185 | 0.395 |
| 2 | Biological structure | 0.000 | 0.000 | 0.000 | 0.218 | 0.116 | nd |
| S3 | Biogeochemical processes | 0.000 | 0.000 | 0.000 | 0.058 | 0.399 | nd |
| S3 | Biological structure | 0.000 | 0.000 | 0.024 | 0.347 | 0.276 | nd |
