# Supplementary material for: Structural and Functional Loss in Restored Wetland Ecosystems
Source: PLoS Biol. 2012 Jan 24;10(1):e1001247. doi: 10.1371/journal.pbio.1001247 (PMC3265451; doi:10.1371/journal.pbio.1001247)
Supplement: Table S5 — Statistical significance of differences between the response ratios in restored or created wetlands under different environmental settings. (DOC) [file pbio.1001247.s009.doc]

**Table S5. Statistical significance of differences between the means of the response ratios in restored or created wetlands under different environmental settings at each age class in years (Kruskal-Wallis test)(nd = no data available, Biol. Struct. = Biological structures, Biogeo. = Biogeochemical processes, * this age class comprises 10 years starting at the beginning of the interval and ending at the end of the interval of the next age class).**

|  |  | **Years since restoration or creation (*p* values)** | | | | | | | |
| --- | --- | --- | --- | --- | --- | --- | --- | --- | --- |
| **Figure** | **Described variable** | **0-5** | **5.1-10** | **10.1-15** | **15.1-20** | **20.1-25** | **25.1-30** | **30.1-35** | **50-55** |
| 3A | Biogeo. seasonal tropical vs. humid temp. | 0.203 | 0.040 | 0.620 | nd | nd | nd | nd | nd |
| 3A | Biogeo. seasonal tropical vs. humid cold | 0.064 | 0.012 | 0.316 | nd | nd | nd | nd | nd |
| 3A | Biogeo. humid temp. vs. humid cold | 0.097 | 0.351 | 0.269 | nd | nd | 0.270 | nd | nd |
| 3B | Biol. struct. seasonal tropical vs. humid temp. | 0.002 | 0.000 | nd | nd | nd | nd | nd | nd |
| 3B | Biol. struct. seasonal tropical vs. humid cold | 0.009 | 0.351 | nd | nd | nd | nd | nd | nd |
| 3B | Biol. struct. seasonal tropical vs. seasonal temp. | 0.000 | nd | nd | nd | nd | nd | nd | nd |
| 3B | Biol. struct. humid temp. vs. humid cold | 0.292 | 0.000 | 0.021 | nd | 0.037* | nd | nd | 0.006 |
| 3B | Biol. struct. humid temp. vs. seasonal temp. | 0.197 | nd | 0.100 | nd | nd | nd | nd | nd |
| 3B | Biol. struct. seasonal temp vs. humid cold | 0.029 | nd | 0.009 | nd | nd | nd | nd | nd |
| 3C | Biogeo. depressional vs. tidal | 0.016 | 0.188 | 0.591 | nd | nd | 0.334 | nd | nd |
| 3C | Biogeo. depressional vs. peatland | 0.129 | nd | nd | nd | nd | 0.339 | nd | nd |
| 3C | Biogeo. depressional vs. riverine | 0.003 | 0.028 | 0.007 | nd | nd | nd | nd | nd |
| 3C | Biogeo. riverine vs. peatland | 0.271 | nd | nd | nd | nd | nd | nd | nd |
| 3C | Biogeo. riverine vs. tidal | 0.371 | 0.214 | 0.009 | 0.378 | nd | nd | nd | nd |
| 3C | Biogeo. tidal vs. peatland | 0.491 | nd | nd | nd | nd | 0.564 | nd | nd |
| 3C | Biol. struct. depressional vs. peatland | 0.886 | 0.913 | nd | nd | nd | nd | nd | 0.504 |
| 3C | Biol. struct. depressional vs. riverine | 0.050 | 0.559 | 0.082 | nd | nd | nd | nd | nd |
| 3C | Biol. struct. depressional vs. tidal | 0.509 | 0.564 | 0.224 | 0.867 | nd | nd | nd | 0.391 |
| 3C | Biol. struct. riverine vs. peatland | 0.475 | 0.722 | nd | nd | nd | nd | nd | nd |
| 3C | Biol. struct. riverine vs. tidal | 0.014 | 0.293 | 0.465 | nd | nd | nd | nd | nd |
| 3C | Biol. struct. tidal vs. peatland | 0.454 | 0.608 | nd | nd | nd | 0.542 | nd | 0.320 |
| S3A | Biogeo. restored vs. created | 0.060 | 0.789 | 0.363 | 0.529 | 0.760 | nd | nd | nd |
| S3B | Biol. struct. restored vs. created | 0.749 | 0.005 | 0.010 | 0.103 | nd | 0.205 | nd | 0.950 |
