# Supplementary material for: Structural and Functional Loss in Restored Wetland Ecosystems
Source: PLoS Biol. 2012 Jan 24;10(1):e1001247. doi: 10.1371/journal.pbio.1001247 (PMC3265451; doi:10.1371/journal.pbio.1001247)
Supplement: Text S1 — Wetland restoration investment and carbon storage calculation. (DOC) [file pbio.1001247.s010.doc]

# Text S1

*Wetland restoration investment*

To estimate total wetland restoration costs, an estimate of the areal proportion of each type (riverine, tidal, and depressional) of restored and created wetlands was calculated from our data-set. These proportions were multiplied by a conservative estimation of the total amount of restored wetlands in North America in the last 20 years (3,000,000 ha)[1], then multiplied by the average price for that type of wetland provided by Zentner et al. 2003 [2]. These cost estimates were updated for 2010 with a 3% annual increase.

*Carbon storage calculation*

From our data-set, we selected only measurements of carbon accumulation (in g C/m^2^, g/cm^3^, g/kg, or % of carbon in soil by weight) in the soil of restored or created and reference wetlands (n=47) excluding restored peatlands. All restored peatlands in our selected studies were not exploited until the mineral soil, thus, maintaining contents of carbon in superficial layers (<20 cm from the soil surface) similar to those in non-impacted reference peatlands. This specific situation would bias our results towards an apparent but unreal recovery. After, we calculated the percentage of carbon lost from the soil by dividing the values at restored and created wetland by the amount of carbon in reference wetland soils. We calculated the average of this value for zero to five years, 5.1 to 10 years, and 10.1 to 25 years after restoration, according with the availability of data-points. Carbon loss of restored and created wetlands was 69% compared to reference wetlands after zero to five years, and 56% after five to 10 years of restoration. Carbon storage and organic matter accumulation are likely correlated variables; however, statistical tests could not be performed because only five studies reported simultaneous measurements of both variables.

*References*

1. Zeug S, Shervette V, Hoeinghaus D, Davisiii S (2007) Nekton assemblage structure in natural and created marsh-edge habitats of the Guadalupe Estuary, Texas, USA. Estuar Coast Shelf Sci 71: 457-466.

2. Matthews JW, Endress AG (2010) Rate of succession in restored wetlands and the role of site context. Applied Vegetation Science: 346-355.
