# Supplementary material for: Structural and Functional Loss in Restored Wetland Ecosystems
Source: PLoS Biol. 2012 Jan 24;10(1):e1001247. doi: 10.1371/journal.pbio.1001247 (PMC3265451; doi:10.1371/journal.pbio.1001247)
Supplement: Text S2 — References used in the meta-analysis. (DOC) [file pbio.1001247.s011.doc]

***Text S2***

***References used in the meta-analysis***

1. Copeland C, A. Zinn J (2008) Wetlands: An Overview of Issues. Washington, DC: Congressional Research Service. RL33483.

2. Zentner J, Glaspy J, Schenk D (2003) Wetland and Riparian Woodland Restoration Costs. Ecol Restor 21: 166-173.

3. Aldous A, McCormick P, Ferguson C, Graham S, Craft C (2005) Hydrologic regime controls soil phosphorus fluxes in restoration and undisturbed wetlands. Restor Ecol 13: 341-347.

4. Armitage AR, Fong P (2004) Gastropod colonization of a created coastal wetland: Potential influences of habitat suitability and dispersal ability. Restor Ecol 12: 391-400.

5. Armitage AR, Jensen SM, Yoon JE, Ambrose RF (2007) Wintering shorebird assemblages and behavior in restored tidal wetlands in southern California. Restor Ecol 15: 139-148.

6. Aronson MFJ, Galatowitsch S (2008) Long-term vegetation developement of restored prairie potholes wetlands. Wetlands 28: 883-895.

7. Ashworth SM (1997) Comparison between restored and reference sedge meadow wetlands in south-central Wisconsin. Wetlands 17: 518-527.

8. Balcombe CK, Anderson JT, Fortney RH, Kordek WS (2005) Wildlife use of mitigation and reference wetlands in West Virginia. Ecol Eng 25: 85-99.

9. Balcombe CK, Anderson JT, Fortney RH, Rentch JS, Grafton WN, et al. (2005) A comparison of plant communities in mitigation and reference wetlands in the mid-appalachians. Wetlands 25: 130-142.

10. Ballantine K, Schneider R (2009) Fifty-five years of soil development in restored freshwater depressional wetlands. Ecol Appl 19: 1467-1480.

11. BishelMachung L, Brooks RP, Yates SS, Hoover KL (1996) Soil properties of reference wetlands and wetland creation projects in Pennsylvania. Wetlands 16: 532-541.

12. Brawley AH, Warren RS, Askins RA (1998) Bird use of restoration and reference marshes within the Barn Island Wildlife Management Area, Stonington, Connecticut, USA. Environ Manage 22: 625-633.

13. Brown SC (1998) Remnant seed banks and vegetation as predictors of restored marsh vegetation. Canadian Journal of Botany-Revue Canadienne De Botanique 76: 620-629.

14. Brown SC (1999) Vegetation similarity and avifaunal food value of restored and natural marshes in northern New York. Restor Ecol 7: 56-68.

15. Brown SC, Smith CR (1998) Breeding season bird use of recently restored versus natural wetlands in New York. J Wildl Manage 62: 1480-1491.

16. Bruland GL, Richardson CJ, Whalen SC (2006) Spatial variability of denitrification potential and related soil properties in created, restored, and paired natural wetlands. Wetlands 26: 1042-1056.

17. Buchsbaum RN, Catena J, Hutchins E, James-Pirri MJ (2006) Changes in salt marsh vegetation, Phragmites australis, and nekton in response to increased tidal flushing in a new England salt marsh. Wetlands 26: 544-557.

18. Castillo JM, Leira-Doce P, Rubio-Casal AE, Figueroa E (2008) Spatial and temporal variations in aboveground and belowground biomass of Spartina maritima (small cordgrass) in created and natural marshes. Estuarine Coastal and Shelf Science 78: 819-826.

19. Cole CA, Brooks RP (2000) A comparison of the hydrologic characteristics of natural and created mainstem floodplain wetlands in Pennsylvania. Ecological Engineering 14: 221-231.

20. Cole CA, Urban CA, Russo P, Murray J, Hoyt D, et al. (2006) Comparison of the long-term water levels of created and natural reference wetlands in northern New York, USA. Ecol Eng 27: 166-172.

21. Cooper DS (2008) The use of historical data in the restoration of the avifauna of the Ballona Wetlands, Los Angeles County, California. Nat Areas J 28: 83-90.

22. Craft C, Broome S, Campbell C (2002) Fifteen years of vegetation and soil development after brackish-water marsh creation. Restor Ecol 10: 248-258.

23. Craft C, Megonigal P, Broome S, Stevenson J, Freese R, et al. (2003) The pace of ecosystem development of constructed Spartina alterniflora marshes. Ecol Appl 13: 1417-1432.

24. Craft C, Reader J, Sacco JN, Broome SW (1999) Twenty-five years of ecosystem development of constructed Spartina alterniflora (Loisel) marshes. Ecological Applications 9: 1405-1419.

25. Craft CB, Seneca ED, Broome SW (1991) Porewater chemistry of natural and created marsh soils. J Exp Mar Biol Ecol 152: 187-200.

26. Dawe NK, Bradfield GE, Boyd WS, Trethewey DEC, Zolbrod AN (2000) Marsh creation in a northern Pacific estuary: Is thirteen years of monitoring vegetation dynamics enough? Conserv Ecol 4.

27. Delphey PJ, Dinsmore JJ (1993) Breeding brid communities of recently restored and natural prairie potholes. Wetlands 13: 200-206.

28. Desrochers DW, Keagy JC, Cristol DA (2008) Created versus natural wetlands: Avian communities in Virginia salt marshes. Ecoscience 15: 36-43.

29. Dodson SI, Lillie RA (2001) Zooplankton communities of restored depressional wetlands in Wisconsin, USA. Wetlands 21: 292-300.

30. Edwards KR, Proffitt CE (2003) Comparison of wetland structural characteristics between created and natural salt marshes in southwest Louisiana, USA. Wetlands 23: 344-356.

31. Fennessy MS, Rokosch A, Mack JJ (2008) Patterns of plant decomposition and nutrient cycling in natural and created wetlands. Wetlands 28: 300-310.

32. Galatowitsch SM (2006) Restoring prairie pothole wetlands: does the species pool concept offer decision-making guidance for re-vegetation? Applied Vegetation Science 9: 261-270.

33. Galatowitsch SM, vanderValk AG (1996) Vegetation and environmental conditions in recently restored wetlands in the prairie pothole region of the USA. Vegetatio 126: 89-99.

34. Galatowitsch SM, vanderValk AG (1996) The vegetation of restored and natural prairie wetlands. Ecol Appl 6: 102-112.

35. Gleason RA, Euliss NH, Hubbard DE, Duffy WG (2004) Invertebrate egg banks of restored, natural, and drained wetlands in the prairie pothole region of the United States. Wetlands 24: 562-572.

36. Graham SA, Craft CB, McCormick PV, Aldous A (2005) Forms and accumulation of soil P in natural and recently restored peatlands - Upper Klamath Lake, Oregon, USA. Wetlands 25: 594-606.

37. Gutrich JJ, Taylor KJ, Fennessy MS (2009) Restoration of vegetation communities of created depressional marshes in Ohio and Colorado (USA): The importance of initial effort for mitigation success. Ecol Eng 35: 351-368.

38. Hampel H, Cattrijsse A, Vincx M (2003) Habitat value of a developing estuarine brackish marsh for fish and macrocrustaceans. ICES J Mar Sci 60: 278-289.

39. Hartzell D, Bidwell JR, Davis CA (2007) A comparison of natural and created depressional wetlands in central Oklahoma using metrics from indices of biological integrity. Wetlands 27: 794-805.

40. Heaven JB, Gross FE, Gannon AT (2003) Vegetation comparison of a natural and a created emergent marsh wetland. Southeast Nat 2: 195-206.

41. Hoeltje SM, Cole CA (2009) Comparison of Function of Created Wetlands of Two Age Classes in Central Pennsylvania. Environ Manage 43: 597-608.

42. Hogan DM, Jordan TE, Walbridge MR (2004) Phosphorus retention and soil organic carbon in restored and natural freshwater wetlands. Wetlands 24: 573-585.

43. Hunter RG, Faulkner SP, Gibson KA (2008) The importance of hydrology in restoration of bottomland hardwood wetland functions. Wetlands 28: 605-615.

44. Janousek CN, Currin CA, Levin LA (2007) Succession of microphytobenthos in a restored coastal wetland. Estuaries and Coasts 30: 265-276.

45. Kellogg CH, Bridgham SD (2002) Colonization during early succession of restored freshwater marshes. Canadian Journal of Botany-Revue Canadienne De Botanique 80: 176-185.

46. Kimball ME, Able KW (2007) Tidal utilization of nekton in Delaware bay restored and reference intertidal salt marsh creeks. Estuaries and Coasts 30: 1075-1087.

47. Lehtinen RM, Galatowitsch SM (2001) Colonization of restored wetlands by amphibians in Minnesota. American Midland Naturalist 145: 388-396.

48. Levin LA, Talley TS (2002) Natural and manipulated sources of heterogeneity controlling early faunal development of a salt marsh. Ecological Applications 12: 1785-1802.

49. Lu JW, Wang HJ, Wang WD, Yin CQ (2007) Vegetation and soil properties in restored wetlands near Lake Taihu, China. Hydrobiologia 581: 151-159.

50. McKenna JE (2003) Community metabolism during early development of a restored wetland. Wetlands 23: 35-50.

51. Melvin SL, Webb JW (1998) Differences in the avian communities of natural and created Spartina alterniflora salt marshes. Wetlands 18: 59-69.

52. Menzel JM, Menzel MA, Kilgo JC, Ford WM, Edwards JW (2005) Bat response to Carolina bays and wetland restoration in the southeastern US Coastal Plain. Wetlands 25: 542-550.

53. Meyer CK, Baer SG, Whiles MR (2008) Ecosystem recovery across a chronosequence of restored wetlands in the platte river valley. Ecosystems 11: 193-208.

54. Meyer CK, Whiles MR (2008) Macroinvertebrate communities in restored and natural Platte River slough wetlands. J N Am Benthol Soc 27: 626-639.

55. Moseman SM, Levin LA, Currin C, Forder C (2004) Colonization, succession, and nutrition of macrobenthic assemblages in a restored wetland at Tijuana Estuary, California. Estuarine Coastal and Shelf Science 60: 755-770.

56. Moser K, Ahn C, Noe G (2007) Characterization of microtopography and its influence on vegetation patterns in created wetlands. Wetlands 27: 1081-1097.

57. Moser KF, Ahn C, Noe GB (2009) The Influence of Microtopography on Soil Nutrients in Created Mitigation Wetlands. Restor Ecol 17: 641-651.

58. Moy LD, Levin LA (1991) Are spartina marshes a replicable resource? - A functional approach to evaluation of marsh creation efforts. Estuaries 14: 1-16.

59. Nair VD, Graetz DA, Reddy KR, Olila OG (2001) Soil development in phosphate-mined created wetlands of Florida, USA. Wetlands 21: 232-239.

60. Neff KP, Rusello K, Baldwin AH (2009) Rapid Seed Bank Development in Restored Tidal Freshwater Wetlands. Restor Ecol 17: 539-548.

61. Paller MH, Reichert MJM, Dean JM, Seigle JC (2000) Use of fish community data to evaluate restoration success of a riparian stream. Ecol Eng 15: S171-S187.

62. Parikh A, Gale N (1998) Vegetation monitoring of created dune swale wetlands, Vandenberg Air Force Base, California. Restor Ecol 6: 83-93.

63. Parkinson RW, DeLaune RR, Hutcherson CT, Stewart J (2006) Tuning surface water management and wetland restoration programs with historic sediment accumulation rates: Merritt Island National Wildlife Refuge, East-Central Florida, USA. J Coast Res 22: 1268-1277.

64. Pechmann JHK, Estes RA, Scott DE, Gibbons JW (2001) Amphibian colonization and use of ponds created for trial mitigation of wetland loss. Wetlands 21: 93-111.

65. Petranka JW, Harp EM, Holbrook CT, Hamel JA (2007) Long-term persistence of amphibian populations in a restored wetland complex. Biol Conserv 138: 371-380.

66. Petranka JW, Kennedy CA, Murray SS (2003) Response of amphibians to restoration of a southern appalachian wetland: A long-term analysis of community dynamics. Wetlands 23: 1030-1042.

67. Petranka JW, Murray SS, Kennedy CA (2003) Responses of amphibians to restoration of a southern appalachian wetland: Perturbations confound post-restoration assessment. Wetlands 23: 278-290.

68. Poach ME, Faulkner SP (1998) Soil phosphorus characteristics of created and natural wetlands in the Atchafalaya Delta, LA. Estuarine Coastal and Shelf Science 46: 195-203.

69. Ratti JT, Rocklage AM, Giudice JH, Garton EO, Golner DP (2001) Comparison of avian communities on restored and natural wetlands in North and South Dakota. J Wildl Manage 65: 676-684.

70. Rozas LP, Minello TJ (2001) Marsh terracing as a wetland restoration tool for creating fishery habitat. Wetlands 21: 327-341.

71. Seabloom EW, van der Valk AG (2003) Plant diversity, composition, and invasion of restored and natural prairie pothole wetlands: Implications for restoration. Wetlands 23: 1-12.

72. Sidle WC, Roose DL, Yzerman VT (2000) Isotope evaluation of nitrate attenuation in restored and native riparian zones in the Kankakee watershed, Indiana. Wetlands 20: 333-345.

73. Stanczak M, Keiper JB (2004) Benthic invertebrates in adjacent created and natural wetlands in northeastern Ohio, USA. Wetlands 24: 212-218.

74. Stevens CE, Diamond AW, Gabor TS (2002) Anuran call surveys on small wetlands in Prince Edward Island, Canada restored by dredging of sediments. Wetlands 22: 90-99.

75. Stevens CE, Gabor TS, Diamond AW (2003) Use of restored small wetlands by breeding waterfowl in Prince Edward Island, Canada. Restor Ecol 11: 3-12.

76. Stolt MH, Genthner MH, Daniels WL, Groover VA, Nagle S, et al. (2000) Comparison of soil and other environmental conditions in constructed and adjacent palustrine reference wetlands. Wetlands 20: 671-683.

77. Streever WJ, Portier KM, Crisman TL (1996) A comparison of dipterans from ten created and ten natural wetlands. Wetlands 16: 416-428.

78. Watts CH, Clarkson BR, Didham RK (2008) Rapid beetle community convergence following experimental habitat restoration in a mined peat bog. Biol Conserv 141: 568-579.

79. Williams GD, Zedler JB (1999) Fish assemblage composition in constructed and natural tidal marshes of San Diego Bay: Relative influence of channel morphology and restoration history. Estuaries 22: 702-716.

80. Zampella RA, Laidig KJ (2003) Functional equivalency of natural and excavated coastal plain ponds. Wetlands 23: 860-876.

81. Zheng L, Stevenson RJ, Craft C (2004) Changes in benthic algal attributes during salt marsh restoration. Wetlands 24: 309-323.

82. Able K, Nemerson D (2004) Evaluating salt marsh restoration in Delaware Bay: analysis of fish response at former salt hay farms. Estuaries and Coasts 27: 58-69.

83. Andersen R, Francez a, Rochefort L (2006) The physicochemical and microbiological status of a restored bog in Québec: Identification of relevant criteria to monitor success. Soil Biol Biochem 38: 1375-1387.

84. Andersen R, Grasset L, Thormann MN, Rochefort L, Francez A-J (2010) Changes in microbial community structure and function following Sphagnum peatland restoration. Soil Biol Biochem 42: 291-301.

85. Bosire JO, Dahdouh-Guebas F, Kairo JG, Koedam N (2003) Colonization of non-planted mangrove species into restored mangrove stands in Gazi Bay, Kenya. Aquat Bot 76: 267-279.

86. Boyer KE, Callaway JC, Zedler JB (2000) Evaluating the Progress of Restored Cordgrass (Spartinafoliosa) Marshes: Belowground Biomass and Tissue Nitrogen. Estuaries 23: 711-721.

87. Byers SE, Chmura GL (2007) Salt Marsh Vegetation Recovery on the Bay of Fundy. Estuaries and coasts 30: 869-877.

88. Card SM, Quideau Sa (2010) Microbial community structure in restored riparian soils of the Canadian prairie pothole region. Soil Biol Biochem 42: 1463-1471.

89. Card SM, Quideau Sa, Oh S-W (2010) Carbon Characteristics in Restored and Reference Riparian Soils. Soil Sci Soc Am J 74: 1834.

90. Chamberlain RH, Beach WP, Barnhart RA (1993) Early Use by Fish of a Mitigation Salt Marsh, Humboldt Bay, California. Estuaries 16: 769-783.

91. Collins BD, Montgomery DR (2002) Forest development, wood jams, and restoration of floodplain rivers in the Puget Lowland, Washington. Restor Ecol 10: 237-247.

92. Elsey-Quirk T, Middleton Ba, Proffitt CE (2009) Seed Dispersal and Seedling Emergence in a Created and a Natural Salt Marsh on the Gulf of Mexico Coast in Southwest Louisiana, U.S.A. Restor Ecol 17: 422-432.

93. Erfanzadeh R, Garbutt A, Pétillon J, Maelfait J-P, Hoffmann M (2009) Factors affecting the success of early salt-marsh colonizers: seed availability rather than site suitability and dispersal traits. Plant Ecol 206: 335-347.

94. Fearnley S (2008) The Soil Physical and Chemical Properties of Restored and Natural Back-Barrier Salt Marsh on Isles Dernieres, Louisiana. J Coast Res 241: 84-94.

95. Fell PE, Murphy KA, Peck MA, Recchia ML (1991) Re-establishment of Melampus bidentatus (Say) and other macroinvertebrates on a restored impounded tidal marsh: comparison of populations above and below the impoundment dike. J Exp Mar Biol Ecol 152: 33-48.

96. Glatzel S (2003) Dissolved organic matter properties and their relationship to carbon dioxide efflux from restored peat bogs. Geoderma 113: 397-411.

97. Hagan SM, Brown SA, Able KW (2007) Production of mummichog (Fundulus heteroclitus): response in marshes treated for common reed (Phragmites australis) removal. Wetlands 27: 54-67.

98. Howe E, Simenstad C (2007) Restoration trajectories and food web linkages in San Francisco Bay’s estuarine marshes: a manipulative translocation experiment. Mar Ecol Prog Ser 351: 65-76.

99. James-Pirri M (2001) Diet Composition of Mummichogs, Fundulus heteroclitus, from Restoring and Unrestricted Regions of a New England (U.S.A.) Salt Marsh. Estuar Coast Shelf Sci 53: 205-213.

100. Kimball ME, Able KW, Grothues TM (2010) Evaluation of Long-Term Response of Intertidal Creek Nekton to Phragmites australis (Common Reed) Removal in Oligohaline Delaware Bay Salt Marshes. Restor Ecol 18: 772-779.

101. Laggoun-Défarge F, Mitchell E, Gilbert D, Disnar J-R, Comont L, et al. (2008) Cut-over peatland regeneration assessment using organic matter and microbial indicators (bacteria and testate amoebae). J Appl Ecol 45: 716-727.

102. Levin LA, Talley D, Thayer G (1996) Succession of macrobenthos in a created salt marsh. Mar Ecol Prog Ser 141: 67-82.

103. Llanso RJ, Bell SS, Vose FE (1998) Food habits of red drum and spotted seatrout in a restored mangrove impoundment. Estuaries 21: 294-306.

104. Luo ZK, Sun OJ, Xu HL (2010) A comparison of species composition and stand structure between planted and natural mangrove forests in Shenzhen Bay, South China. Journal of Plant Ecology-Uk 3: 165-174.

105. Macintosh DJ, Ashton EC, Havanon S (2002) Mangrove rehabilitation and intertidal biodiversity: A study in the Ranong mangrove ecosystem, Thailand. Estuarine Coastal and Shelf Science 55: 331-345.

106. Marchetti MP, Garr M, Smith ANH (2008) Evaluating Wetland Restoration Success Using Aquatic Macroinvertebrate Assemblages in the Sacramento Valley, California. Restor Ecol 18: 457-466.

107. McKee KL, Faulkner PL (2000) Restoration of biogeochemical function in mangrove forests. Restor Ecol 8: 247-259.

108. Meyer CK, Whiles MR, Baer SG (2010) Plant Community Recovery following Restoration in Temporally Variable Riparian Wetlands. Restor Ecol 18: 52-64.

109. Miller MJ, Able KW (2004) Movements and growth of tagged young-of-the-year Atlantic croaker ( Micropogonias undulatus L .) in restored and reference marsh creeks in Delaware. J Exp Mar Biol Ecol 267: 15 - 33.

110. Minello TJ, Zimmerman RJ (1992) Utilization of natural and transplanted Texas salt marshes by fish and decapod crustaceans. Mar Ecol Prog Ser 90: 273-285.

111. Peck MA, Fell PE, Allen EA, Gieg JA, Guthke CR, et al. (1994) Evaluation of tidal marsh restoration: comparison of selected macroinvertebrate populations on a restored impounded valley marsh and an unimpounded valley marsh within the same salt marsh system in Connecticut, USA. Environ Manage 18: 283-293.

112. Piehler MF, Currin CA, Cassanova R, Paerl HW (1998) Development and N_2_-Fixing Activity of the Benthic Microbial Community in Transplanted Spartina alterniflora Marshes in. Restor Ecol 6: 290-296.

113. Raposa K (2002) Early Responses of Fishes and Crustaceans to Restoration of a Tidally Restricted New England Salt Marsh. Restor Ecol 10: 665-676.

114. Raposa KB (2008) Early Ecological Responses to Hydrologic Restoration of a Tidal Pond and Salt Marsh Complex in Narragansett Bay , Rhode Island. J Coast Res 55: 180-192.

115. Ren H, Jian SG, Lu HF, Zhang QM, Shen WJ, et al. (2008) Restoration of mangrove plantations and colonisation by native species in Leizhou bay, South China. Ecol Res 23: 401-407.

116. Rozas L, Minello T (2009) Using nekton growth as a metric for assessing habitat restoration by marsh terracing. Mar Ecol Prog Ser 394: 179-193.

117. Rozas LP, Minello TJ (2007) Restoring coastal habitat using marsh terracing: the effect of cell size on nekton use. Wetlands 27: 595-609.

118. Rozas LP, Minello TJ, Zimmerman RJ, Caldwell P (2007) Nekton populations, long-term wetland loss, and the effect of recent habitat restoration in Galveston Bay, Texas, USA. Marine Ecology-Progress Series 344: 119-130.

119. Teo S (2003) Growth and production of the mummichog (*Fundulus heteroclitus*) in a restored salt marsh. Estuaries and Coasts 26: 51-63.

120. Thompson SP, Paerl HW, Go MC (1995) Seasonal patterns of nitrification and denitrification in a natural and a restored salt marsh. Estuaries 18: 399-408.

121. Tupper M, Able KW (2000) Movements and food habits of striped bass (*Morone saxatilis*) in Delaware Bay ( USA ) salt marshes : comparison of a restored and a reference marsh. Mar Biol: 1049-1058.

122. Vose FE, Bell SS (1994) Resident fishes and macrobenthos in mangrove-rimmed habitats: Evaluation of habitat restoration by hydrological modification. Estuaries 17: 585-596.

123. Waddington JM, Warner KD (2001) Atmospheric CO_2_ sequestration in restored mined peatlands. Ecoscience 8: 359.

124. Walton ME, Le Vay L, Lebata JH, Binas J, Primavera JH (2007) Assessment of the effectiveness of mangrove rehabilitation using exploited and non-exploited indicator species. Biol Conserv 138: 180-188.
